# To the Editors of the Medical and Physical Journal

**Published:** 1804-04-01

**Authors:** 


### *To the Editors of the Medical and Physical Journal.*

GENTLEMEN,

ABOUT two months ago a celebrated Professor * at one of our medical schools, announced an important discovery, which I have since been anxiously waiting to see communicated to the public; but as the knowledge of it appears either very confined, or men of science very tardy in forwarding its best interests, I shall, with your permission, state it as concisely as I can. He observed, that a learned Orientalist had in his possession an Arabic manuscript, which traces the origin of the small-pox to the same source from which Dr. Jenner has satisfactorily demonstrated the vaccine disease to proceed.

The Arabians have, in all ages, been famed for their horses and camels, and the milk of the latter constitutes a part of their subsistence. Thus the virus from the diseased heel of the horse is transferred to the papillæ of the camel, in the same manner as it is to those of the cow; and the camel-pox is produced, the poison of which, insinuating itself by some slight wound into the human system, the small-pox, with all its attendant horrors, is the consequence.

Should this fact be authenticated, it will attract the attention of naturalists to the hitherto inexplicable causes of contagious diseases; and many that have eluded the scrutinizing eye of sagacity for ages, will probably be devoloped and brought to light; analogies will be discovered of which we have now no idea; and antidotes that may perhaps arrest the progress of some of the most dreadful scourges of humanity.

If any of your readers are acquainted with any further particulars relative to this very interesting subject, they would certainly promote the cause of science by communicating them to the public through the medium of the Medical Journal. Those to whom it may be no novelty can perhaps inform us to whom the manuscript belongs, and whether (as was intimated) a translation of it is preparing for the press. I am, &c.

*B. S. Feb.* 2, 1804. STUDIOSUS.

* Mr. Abernethy.

